# Physical function and associations with diet and exercise: Results of a cross-sectional survey among elders with breast or prostate cancer

**DOI:** 10.1186/1479-5868-1-16

**Published:** 2004-10-29

**Authors:** Wendy Demark-Wahnefried, Elizabeth C Clipp, Miriam C Morey, Carl F Pieper, Richard Sloane, Denise Clutter Snyder, Harvey J Cohen

**Affiliations:** 1 Department of Surgery, Duke University Medical Center (DUMC), Durham, USA; 2 School of Nursing, DUMC, Durham, USA; 3 Program of Cancer Prevention, Detection & Control Research, DUMC, Durham, USA; 4 Older Americans Independence Center/Center for Aging & Human Development, DUMC, Durham, USA; 5 Department of Medicine, DUMC, Durham, USA; 6 Geriatric Research, Education and Clinical Center, VA Medical Center, Durham, USA; 7 Department of Biometry and Bioinformatics, DUMC, Durham, USA

## Abstract

**Background:**

Functional decline threatens independent living and is common among individuals diagnosed with cancer, especially those who are elderly. The purpose of this study was to explore whether dietary and exercise practices are associated with physical function status among older cancer survivors.

**Methods:**

Mailed surveys were used to ascertain data on physical function, dietary fat, fruit and vegetable (F&V) consumption, and exercise among elderly diagnosed with early stage (I-II) breast (N = 286) or prostate cancer (N = 402) within the past 18 months.

**Results:**

Sixty-one percent of respondents reported diets with <30% of energy from fat, 20.4% reported F&V intakes of 5+ daily servings, and 44.6% reported regular vigorous exercise. Significant, independent associations were found between physical functioning and reported dietary fat intake, F&V consumption, and exercise. A simultaneous multiple regression model controlled for age, race, gender, time since diagnosis and concurrent health behaviors yielded the following estimates: (1) 0.2 increase in the SF-36 physical function subscale (PFS) score with each reported 1% decrease in percent energy from fat (p < .0001); (2) 0.9 increase in the SF-36 PFS score for each reported serving of F&V/day (p = .0049); and (3) 15.4 increase in the SF-36 PFS score with a positive response for regular vigorous exercise (p < .0001).

**Conclusions:**

Results of this cross-sectional survey suggest that regular vigorous exercise and consumption of diets low in fat and rich in F&Vs are associated with higher levels of physical functioning among older cancer survivors. Interventions that promote healthful lifestyle change may deliver considerable benefit within this ever increasing and vulnerable population.

## Background

There are 9.6 M cancer survivors in the US today, and 61% are age 65 or older [1]. Over the next 50 years, the number of elderly cancer survivors is expected to double [2]. Unfortunately, these individuals are at greater risk for other cancers, cardiovascular disease, osteoporosis, diabetes, and functional decline [1-5]. While all elderly are at increased risk for functional decline [6], those diagnosed with cancer are even more vulnerable and may experience functional losses that threaten independent living [7-10]. Adherence to healthful lifestyle behaviors may be one way that older cancer survivors can maintain or regain higher levels of physical functioning. Yet, there are little data to support this premise.

## Methods

We explored associations between lifestyle factors and physical functioning among elderly cancer survivors who were screened for Project LEAD (Leading the Way in Exercise And Diet), a home-based, diet and exercise intervention trial [11]. Briefly, individuals (age 60+) who were no more than 18 months beyond a diagnosis of early stage (I-II) breast or prostate cancer were identified through cancer registries, private practices, and self-referral. Patients were mailed a letter of invitation, consent forms, a postage-paid return envelope, and a brief survey. The purpose of the survey was to screen-out individuals already practicing healthful behaviors [i.e., those engaging in regular, vigorous exercise or consuming a low fat diet with 5 or more daily servings of fruits and vegetables (F&V)] and individuals unlikely to benefit from the telephone counseling-mailed material intervention [i.e., those who were mentally incompetent, severely hearing impaired, or who had conditions that precluded unsupervised exercise or a high F&V diet (i.e., scheduled hip or knee replacement, walker or wheelchair use, recent stroke or heart attack, angina, congestive heart failure, chronic obstructive pulmonary disease, end-stage renal failure, and/or warfarin-use)]. The survey incorporated validated scales to assess dietary fat (Block Dietary Fat Screener) [12], and F&V consumption (5 A Day items) [13]. Given space constraints of the survey and our focus on vigorous exercise, one item was used to assess exercise ("On average, do you do continuous vigorous exercise for at least 20 minutes, 3 or more times per week?") [14,15]. The SF-36 Physical Function Subscale (SF-36 PFS) also was modified slightly to omit item #2 ("Does your health limit you in moderate activities") and scores were scaled 0–100 with the assumption that the average value for the missing item was the same as that for the remaining items [16].

T-tests and chi-square analyses were performed to determine if respondents differed from non-respondents on age, time since diagnosis, race and gender, and to test for differences between male and female respondents. Pearson correlation coefficients indicated associations between F&V intake and percent dietary fat, and physical function scores. T-tests were used to explore associations between exercise and function. Linear regression analyses permitted an examination of associations between physical function and health behaviors controlling for several likely confounders including gender, age, race, time since diagnosis and concurrent health behaviors. Given the homogeneity in stage at diagnosis, stage information was omitted from this analysis.

## Results and Discussion

Between August 2000 and May 2003, 2,431 cases were identified and physician approval for contact was granted for 2,034 cases. Incomplete address information existed for 24 of these cases, yielding 2,010 viable posted surveys, of which 688 complete surveys were returned (34% response rate). Characteristics of respondents and non-respondents are provided in Table 1. Respondents were significantly more likely than non-respondents to be younger, white, male, and more proximal to their date of diagnosis.

**Table 1:** Demographic characteristics of respondents and non-respondents

Characteristic	Respondents (n = 688)	Non-Respondents (n = 1322)	P-value
Age (years)			
Mean (sd)	71.4 (5.0)	73.0 (5.9)	<.0001
Range	60 – 94	64 – 96	
Race [% (N)]			
White	83.4% (574)	75.0% (991)	<.0001
African American	12.4% (85)	21.0% (278)	
Other/Unknown	4.2% (29)	4.0% (53)	
Gender [% (N)]			
Female	41.6% (286)	52.7% (696)	<.0001
Male	58.4% (402)	47.4% (626)	
Time Elapsed Since Diagnosed (months)			
Mean (sd)	10.8(4.9)	11.3 (5.8)	.046
Distribution [%/(N)]			
0 – 3 months post-diagnosis	9.2% (63)	11.4% (151)	
>3 – 6 months post-diagnosis	8.9% (61)	10.1% (134)	
>6 – 9 months post-diagnosis	24.0% (165)	17.4% (230)	
>9 – 12 months post-diagnosis	21.8% (150)	20.7% (274)	
>12 – 15 months post-diagnosis	18.0% (124)	15.9% (210)	
15+ months post-diagnosis	18.2% (125)	24.4% (323)	

Data regarding physical function and lifestyle behaviors are provided in Table 2. In bivariate analyses, elders with prostate cancer (men) have significantly higher SF-36 PFS scores than those with breast cancer (women). A majority of all respondents adhere to a low fat diet, while a minority pursue regular vigorous exercise or eat 5 or more daily servings of F&Vs. Men are significantly more likely than women to exercise and to consume 5 or more daily servings of F&Vs. However, female cancer survivors are more likely to report low fat diets.

**Table 2:** Physical function and health behaviors of survey respondents (N = 688)

Variable	Total Sample (N = 688)	Women with Breast Cancer (N = 286)	Men with Prostate Cancer (N = 402)	p-value
Physical Function				
[SF-36 – 9 items scaled 0–100, mean (sd)]	74.3 (25.3)	68.3 (25.5)	78.5 (24.3)	<.0001
Exercise				
(% who "vigorously exercise for at least 20 minutes, 3 or more times a week")	44.6%	36.7%	50.2%	.001
% of Energy (Calories) from Fat				
mean (sd)	29.1 (21.8)	25.6 (16.2)	31.7 (24.7)	.0003
% consuming <30% of Energy from Fat	61.1%	65.8%	57.6%	.031
Fruit and Vegetable Consumption				
mean number of servings (sd)	4.1 (2.9)	3.7 (2.3)	4.4 (3.2)	.0005
% consuming 5+ servings/day	20.4%	15.4%	24.1%	.005

Modest correlations were obtained between the indicators of exercise and diet, with positive agreement noted between F&V intake and exercise (Pearson ρ = .12/p = .003) as well as F&V intake and dietary fat (ρ = .42/p < .0001). An inverse association was noted between dietary fat and exercise (ρ = -.08/p = .05). In bivariate associations, all three behaviors were significantly associated with SF-36 physical functioning scores (p < .05): F&V intake (ρ = .09), dietary fat (ρ = -.10), and vigorous exercise (ρ = .37). In multivariable linear regression analyses, with the SF-36 PFS score serving as the dependent variable and controlling for age, race, gender, time since diagnosis, and concurrent health behaviors, the simultaneous associations of the three indicators remained statistically significant (independent) and yielded the following estimates: (1) a 0.2 increase in the SF-36 PFS score with each reported 1% decrease in percent energy from fat (p < .0001); (2) a 0.9 increase in the SF-36 PFS score for each reported serving of F&V/day (p = .0049); and (3) a 15.4 increase in the SF-36 PFS score with a positive response for regular vigorous exercise (p < .0001).

In comparing these cross-sectional data on lifestyle behaviors of older cancer survivors to normative data reported on healthy elders, as well as to previous data reported on general populations of breast and prostate cancer survivors (where a dearth of data have been reported on older cancer survivors per se), we find both differences and similarities. Like data that exist on healthy elders (age 65+) responding to the 2000 Behavioral Risk Factor Surveillance System (BRFSS) survey [17], or findings of a previous study of 978 breast and prostate cancer survivors [18], a minority of the respondents to this survey report consuming 5 or more daily servings of F&Vs. However, the percentage of our respondents who reportedly achieved 5 A Day guidelines was lower than that reported by these two previous studies (20.4% as compared to 34.4% and 42%, respectively) [17,18]. In contrast, a majority of survivors in both this study (61.1%) and the previous study on cancer survivors (69%) report adherence to a low fat diet [18], whereas higher mean intakes of fat 32–33% were noted among a general population of elders (age 60+) responding to the National Health and Nutrition Examination Survey, Phase I (1988–1991) [19]. Like the previous study on breast and prostate cancer survivors (where 58% reported routine exercise of moderate intensity or greater compared to 47% within the general population) [18,20], a greater proportion of these elderly cancer survivors report routine vigorous exercise when compared to BRFSS data on the general population (i.e., 44.6% vs. 11%) [20]. Findings may differ due to differences in survey instruments, populations and varying amounts of respondent bias, however at least for dietary fat and exercise, data largely support the prevalent finding that cancer survivors tend to report healthier lifestyle behaviors [21]. The fact, that we did not see this trend in F&V consumption may be due to non-penetrance of the 5 A Day message among the survivor population, or may be the result of our population being significantly older than that reported in previous studies – a population where proportionally more individuals may be edentulous or suffering from G.I. intolerances that serve as barriers to F&V consumption. Our data on physical function, however, indicate higher levels within our sample (74.3 ± 25.3) when compared to U.S. age-matched norms (69.4 ± 26.3) [22], despite previous findings, which suggest decreased physical functioning among older cancer survivors [7-10].

A potential explanation for the higher physical function scores exhibited within our sample may relate to the higher prevalence of healthful lifestyle practices which in turn may increase physical function. This links back to the primary thrust of this study, which was to determine evidence for a link between functional status and health behavior.

Our results suggest that regular vigorous exercise and a diet rich in F&Vs and low in fat is associated with higher levels of physical functioning among elderly recently diagnosed with cancer. Although the association between exercise and improved function has been reported repeatedly in other studies among elders [6,23], this is the first study to show this association exclusively among elderly cancer survivors. Further, the fact that regular vigorous activity is associated with a 15.4 point differential in functional status, accounts for a magnitude of effect that is comparable to 0.61 standard deviations (sd), and far exceeds the 0.22 sd noted in a previous study by Baker et al. as being both statistically and clinically significant [7].

Furthermore, only one study to date has reported the link between diet composition and physical function. Ortega et al. found that diets low in fat and high in F&Vs were associated with higher levels of physical functioning among a sample of upper socio-economic, elderly male Spaniards at risk for cardiovascular disease [24]. This previous report however did not include defined estimates, so it is difficult to draw exact comparisons regarding the magnitude of dietary change associated with a given difference in SF-36 PFS score. Nonetheless, our data suggest that modest gains in physical functioning may be achieved with dietary changes that are both feasible and realistic [i.e., a 0.9 increase in PFS score with each additional serving of F&V or a 0.2 increase in PFS score with each 1% decrease in percent energy from fat (roughly equivalent to a half a teaspoon of butter, margarine or oil for most caloric intakes)]. Albeit, multiple servings of F&Vs (roughly 5 per day) or substantial decreases in fat (approximately 20% of total Calories) would be necessary to achieve clinical significance if dietary changes were pursued in isolation, however if taken together, as in the pursuit of a healthier overall diet, it is conceivable that appreciable functional improvement could occur. While the relationship between exercise and physical function is strong and mechanistically more direct, low fat diets (with lower amounts of saturated and trans fats) and increased F&Vs also provide theoretically viable, yet not fully elucidated pathways, to increased function [25]. It must be noted, however that our findings differ somewhat from those of a recently reported study of cancer survivors by Blanchard et al. [26], who found a significant association between Health Related Quality of Life (HRQOL) and exercise, but not between HRQOL and F&V intake. Differences between studies with regard to the sample (e.g. our sample included 688 elderly breast and prostate cancer survivors whereas the sample of Blanchard et al. [26] was comprised of 316 breast, prostate and colorectal cancer survivors of all ages), and survey items (e.g., we used validated items taken from the National 5 a Day trials [13], whereas Blanchard et al. [26] used one composite item) may account for the discrepancy in findings. Accordingly, and for the purposes of designing effective interventions to improve physical function in older cancer survivors, further research is necessary to corroborate associations between diet and physical function (if any) and to clarify responsible mechanisms.

The primary limitations of this study relate to respondent bias and cross-sectional design. Our response rate of 34% is indeed less than the 59% we have achieved in previous mailed surveys among similar populations [18]. Given that this survey was linked to accrual efforts for a yearlong behavioral intervention trial [11], a response rate in this range was anticipated. Indeed, our response rate is similar to those of 35–50% cited by Martin Brown, PhD (Chief of the National Cancer Institute's Health Services and Economics Branch) in a published interview regarding survey responses rates among cancer patients [27]. In addition, in attempting to control for factors that differed between respondents and non-respondents, we acknowledge that there likely exist other important factors that were not included (e.g. items that our survey did not ascertain such as socio-economic status), or the fact that we were unable to assess and subsequently control for lifestyle behaviors among those who did not respond – non-respondents whose lifestyle behaviors may have placed them at risk (i.e., sedentary, poor diets) and less inclined to respond, or conversely those already adhering to healthy lifestyle behaviors and less compelled to participate. Another limitation of our study was the use of abbreviated scales or items to obtain health-related data. As an example, the single item used to capture vigorous exercise may have led to inflated rates of reporting due to the absence of a response category for moderate exercise. Finally, our study was cross-sectional, and cause and effect possibly are confounded.

## Conclusions

Findings of this study support the recent American Cancer Society diet and exercise guidelines for survivors [25], which call for a physically active lifestyle and a plant-based diet. However, more research is needed to confirm associations between lifestyle factors and physical function, especially among broader populations of survivors (i.e., other types of cancer, other age groups, and among short-term versus long-term survivors). Ultimately, randomized controlled trials enrolling of older persons with cancer are needed to determine the potential benefit of diet and exercise interventions in reorienting the trajectory of functional decline.

## List of Abbreviations

F&V: Fruits and Vegetables

SF-36: Short Form-36 Health Survey

PFS: Physical Function Subscale

## Competing Interests

The author(s) declare that they have no competing interests.

## Authors' Contributions

WDW, ECC, MCM, CFP and HJC conceived of the study, and participated in its design. DCS participation in the coordination of the study, as well as data cleaning and interpretation of findings. RS performed the statistical analysis. All authors read and approved the final manuscript.
